# Genetic and Phylogenetic Characterization of Tataguine and Witwatersrand Viruses and Other Orthobunyaviruses of the Anopheles A, Capim, Guamá, Koongol, Mapputta, Tete, and Turlock Serogroups

**DOI:** 10.3390/v7112918

**Published:** 2015-11-23

**Authors:** Alexey M. Shchetinin, Dmitry K. Lvov, Petr G. Deriabin, Andrey G. Botikov, Asya K. Gitelman, Jens H. Kuhn, Sergey V. Alkhovsky

**Affiliations:** 1 D.I. Ivanovsky Institute of Virology, Gamaleya Federal Research Center for Epidemiology and Microbiology, Ministry of Health of the Russian Federation, 123098, Moscow, Russia; shchetinin.alexey@yandex.ru (A.M.S.); dk_lvov@mail.ru (D.K.L.); pg_deryabin@mail.ru (P.G.D.); tessey@mail.ru (A.G.B.); gitelman_ak@mail.ru (A.K.G.); 2 Integrated Research Facility at Fort Detrick, National Institute of Allergy and Infectious Diseases, National Institutes of Health, Fort Detrick, Frederick, MD 21702, USA; kuhnjens@mail.nih.gov

**Keywords:** Anopheles A virus, bunyavirus, Capim virus, Guamá virus, Koongol virus, orthobunyavirus, Tataguine virus, Tete virus, Turlock virus, Witwatersrand virus

## Abstract

The family *Bunyaviridae* has more than 530 members that are distributed among five genera or remain to be classified. The genus *Orthobunyavirus* is the most diverse bunyaviral genus with more than 220 viruses that have been assigned to more than 18 serogroups based on serological cross-reactions and limited molecular-biological characterization. Sequence information for all three orthobunyaviral genome segments is only available for viruses belonging to the Bunyamwera, Bwamba/Pongola, California encephalitis, Gamboa, Group C, Mapputta, Nyando, and Simbu serogroups. Here we present coding-complete sequences for all three genome segments of 15 orthobunyaviruses belonging to the Anopheles A, Capim, Guamá, Kongool, Tete, and Turlock serogroups, and of two unclassified bunyaviruses previously not known to be orthobunyaviruses (Tataguine and Witwatersrand viruses). Using those sequence data, we established the most comprehensive phylogeny of the *Orthobunyavirus* genus to date, now covering 15 serogroups. Our results emphasize the high genetic diversity of orthobunyaviruses and reveal that the presence of the small nonstructural protein (NSs)-encoding open reading frame is not as common in orthobunyavirus genomes as previously thought.

## 1. Introduction

The family *Bunyaviridae* ranks among the largest families of RNA viruses. The family has more than 530 named members that are either assigned to the five included genera *Hantavirus*, *Nairovirus*, *Orthobunyavirus*, *Phlebovirus*, or *Tospovirus*, or remain to be classified [1,2,3]. Many bunyaviruses can cause disease in humans. These diseases commonly manifest as arthritides/rashes, fevers/myalgias, pulmonary diseases, encephalitides, or viral hemorrhagic fevers [4]. In addition, bunyaviruses can cause severe disease in wild and domesticated animals and wild or cultivated crop or ornamental plants (tospoviruses only). The majority of bunyaviruses is transmitted among hosts by arthropods (predominantly mosquitoes, ticks, sandflies, biting midges, or thrips), whereas hantaviruses are transmitted by contaminated excreta or secreta or bites of infected rodents, eulipotyphla, or bats [1,3]. In addition, several genus-level clades of insect-specific bunyaviruses (Ferak and Jonchet virus clades, “goukoviruses”, “herbeviruses”, “phasmaviruses”) have been described recently. Among those, “herbeviruses,” *i.e.*, Herbert virus, Kibale virus, and Taï virus, form a deeply-rooted sister taxon to the genus *Orthobunyavirus* [5,6].

Bunyaviruses have tripartite, negative-sense, single-stranded RNA genomes [7,8]. The three linear genomic segments, commonly referred to as small (S), medium (M), and large (L) based on their overall lengths, are characterized by complementary 3′- and 5′-untranslated regions (UTRs) that form panhandle-like structures. The S segment encodes the nucleocapsid protein (N) and often a small nonstructural protein (NSs). The M segment encodes a glycoprotein precursor polyprotein, which after cleavage yields viral glycoproteins (Gn and Gc) and, in the case of some bunyaviruses, a small, nonstructural protein (NSm). The L segment encodes the RNA-dependent RNA polymerase (RdRp, L). The glycoproteins mediate virion entry into susceptible cells through endocytosis. L transcribes and replicates the individual segments in the cytosol. Newly synthesized N encapsidates progeny RNA genome segments to form ribonucleoprotein complexes that associate with a few molecules of RdRp. These complexes acquire glycoprotein-containing envelopes upon budding into Golgi-derived vesicles and egress from the host cell by exocytosis. The functions of the various nonstructural proteins are less clear, but NSs has been identified as an interferon antagonist [7,8].

The genus *Orthobunyavirus* is the most complex genus of the family *Bunyaviridae* and currently contains approximately 220 named viruses. The majority of these viruses were assigned to more than 18 different serogroups based on presence, lack, or extent of serological cross reactions using various assays, such as complement fixation (CF), hemagglutination inhibition (HI), and neutralization test (NT) [9,10,11,12,13,14]. Mostly within those serogroups, these orthobunyaviruses were assigned to 48 official species [2]. Until recently, genomic sequence information was limited to some orthobunyaviruses of the Bunyamwera, California encephalitis, Simbu, and Group C serogroups [15,16,17,18,19,20,21,22,23]. Recent advances in next-generation sequencing have led to an acquisition of coding-complete and sometimes complete genome sequences of some orthobunyaviruses from the Bwamba/Pongola, Gamboa, Mapputta, and Nyando serogroups, and of several ungrouped viruses [24,25,26,27,28]. However, viruses of more than 10 serogroups remain to be characterized on the genomic level.

Here we report the first coding-complete genomic sequences of 15 distinct orthobunyaviruses from the Anopheles A, Capim, Guamá, Koongol, Mapputta, Tete, and Turlock serogroups (Table 1).

Viruses of the Anopheles A serogroup have been isolated from *Aedes* and *Anopheles* mosquitoes mostly collected in South American and Caribbean countries. Eleven different viruses of this group have been assigned to the two species *Anopheles A orthobunyavirus* (Anopheles A, Arumateua, Caraipé, Las Maloyas, Lukuni, Trombetas, Tucuruí viruses) and *Tacaiuma orthobunyavirus* (CoAr 1071, CoAr 3627, Tacaiuma, Virgin River viruses) [1,2,29,30,31,32]. We present coding-complete sequence information for Lukuni virus, which was originally isolated from *Aedes* mosquitoes collected in 1955 in Trinidad and Brazil [33], but has not been associated with human disease.

Capim serogroup viruses are classified into five species: *Acara orthobunyavirus* (Acara, Moriche viruses), *Benevides orthobunyavirus* (Benevides virus), *Bushbush orthobunyavirus* (Benfica, Bushbush, Juan Diaz viruses), *Capim orthobunyavirus* (Capim virus), and *Guajará orthobunyavirus* (Guajará virus). These viruses are endemic to South and Northern America, where they are transmitted by mosquitoes among small vertebrates [1,2,32]. We determined the coding-complete sequence of Capim virus and Guajará virus. Capim virus was originally isolated from a trapped woolly opossum (*Caluromys philander*) in 1958 in Pará State, Brazil, and was also recovered from sentinel spiny rats (*Proechimys* spp.) and *Culex* mosquitoes. Antibodies to the virus were detected in spiny rat sera, but not in human sera in Pará State. Guajará virus was first isolated from a sentinel Swiss laboratory mouse in Pará State, Brazil. Additional isolates were obtained in South America from wild rodents and mosquitoes of different species, and antibodies against the virus were detected repeatedly in spiny rats [11,34,35].

**Table 1:** Newly sequenced orthobunyaviruses (this study).

Orthobunyavirus Serogroup Virus Name (Virus Abbreviation) Isolate Designation	Year of Virus Isolation	Country of Virus Isolation (Current Country Name)	Virus Source (Species)	Signs of Human Infection	Reference(s)	New GenBank Accession Numbers
**Anopheles A serogroup**						
Lukuni virus (LUKV)						
TRVL 10076	1955	Trinidad and Tobago Crown Colony of the British Empire (Trinidad and Tobago)	Mosquitoes (*Aedes* (*Ochlerotatus*) *scapularis*)	NR	[33]	KP792670-72
**Capim serogroup**						
Capim virus (CAPV)						
BeAn 8582	1958	Brazil	Woolly opossum (*Caluromys philander)*	NR	[34]	KT160026-28
Guajará virus (GJAV)						
BeAn 10615	1959	Brazil	Swiss laboratory mouse, sentinel	NR	[34,35]	KP792661-63
**Guamá serogroup**						
Bimiti virus (BIMV)						
TRVL 8362	1955	Trinidad and Tobago Crown Colony of the British Empire (Trinidad and Tobago)	Mosquitoes (*Culex* (*Melanoconion*) *spissipes*)	NR	[36,37]	KP792655-57
Catú virus (CATUV)						
BeH 151	1955	Brazil	Mosquitoes (*Culex* (*Melanoconion*) *spissipes*)	Fever, myalgia	[38]	KP792658-60
Guamá virus (GMAV)						
BeAn 277	1955	Brazil	Tufted capuchin (*Cebus apella*), sentinel	Fever, myalgia	[38]	KP792664-66
Mahogany Hammock virus (MHV)						
Fe4-2a	1964	USA	Mosquitoes (*Culex* (*Melanoconion*) sp.)	NR	[39]	KP835518-20
Moju virus (MOJUV)						
BeAr 12590	1959	Brazil	Mosquitoes (*Culex* sp.)	NR	[34]	KP792673-75
**Koongol serogroup**						
Koongol virus (KOOV)						
MRM31	1960	Australia	Mosquitoes (*Culex* (*Culex*) *annulirostris*)	NR	[40]	KP792667-69
**Mapputta serogroup**						
Mapputta virus (MAPV)						
MRM186	1960	Australia	Mosquitoes (*Anopheles* (*Cellia*) *meraukensis*)	NR	[40]	KP792694-96
Trubanaman virus (TRUV)						
MRM3630	1966	Australia	Mosquitoes (*Anopheles* (*Cellia*) *annulipes*)	Arthritis, rash	[41,42]	KP792682-84
**Tete serogroup**						
Bahig virus (BAHV)						
EgB 90	1966	Egypt	Eurasian golden oriole (*Oriolus oriolus*)	NR	[43]	KP792652-54
Matruh virus (MTRV)						
An-1047	1961	United Arab Republic (Egypt)	Lesser whitethroat (*Sylvia curruca)*	NR	[43]	KP792691-93
Tete virus (TETEV)						
SAAn 3518	1959	Union of South Africa (South Africa)	Village weaver (*Ploceus cucullatus*)	NR	[44]	KP792679-81
**Turlock serogroup**						
Umbre virus (UMBV)						
IG1424	1955	India	Mosquitoes (*Culex* (*Oculeomyia*) *bitaeniorhynchus*)	NR	[44]	KP792685-87
**Unassigned**						
Tataguine virus (TATV)						
Ib-H 9963	1968	Nigeria	Human (*Homo sapiens*)	Fever, myalgia	[45]	KP792676-78
Witwatersrand virus (WITV)						
SAAr 1062	1958	Union of South Africa (South Africa)	Mosquitoes (*Culex* (*Eumelanomyia*) *rubinotus*)	NR	[46]	KP792688-90

NR, none reported.

The Guamá serogroup viruses are distributed among five established species: *Bertioga orthobunyavirus* (Bertioga, Cananeia, Guaratuba, Itimirim, Mirim viruses), *Bimiti orthobunyavirus* (Bimiti virus), *Catú orthobunyavirus* (Catú virus), *Guamá orthobunyavirus* (Ananindeua, Guamá, Mahogany Hammock, Moju viruses), and *Timboteua orthobunyavirus* (Timboteua virus) [1,2,32]. Guamá serogroup viruses are mostly endemic to South America. The exceptions are Guamá and Mahogany Hammock viruses, which were isolated in North America. Catú virus and Guamá virus were isolated from humans with fever/myalgia. Viruses of the Guamá serogroup are usually transmitted by culicine mosquitoes among vertebrate hosts including bats, birds, marsupials, and rodents [34,36,37,38,39,47]. Here, we present coding-complete sequences for Bimiti, Catú, Guamá, Mahogany Hammock, and Moju viruses.

The Koongol serogroup currently consists of Koongol virus and Wongal virus, both of which are assigned to the species *Koongol orthobunyavirus* [1,2,32]. Both viruses were originally isolated in 1960 in Queensland, Australia, from *Culex* mosquitoes [40]. Koongol virus, newly sequenced here, was also obtained from *Ficalbia* mosquitoes in New Guinea [35]. HI tests suggested that both Koongol and Wongal viruses may be able to infect a range of mammals, marsupials, and possibly birds, and may be widespread throughout Australia. However, these results were not confirmed using NT [48,49,50].

The Mapputta serogroup currently has seven members (Buffalo Creek, Gan Gan, Mapputta, Maprik, Murrumbidgee, Salt Ash, Trubanaman viruses) that have not yet been assigned to species [1,24,51]. Buffalo Creek virus and Murrumbidgee viruses [24,52,53] were isolated from *Anopheles* mosquitoes collected in Northern Territory and Griffith, Australia, respectively. Mapputta and Trubanaman viruses, which we have sequenced during this study, were initially isolated from *Anopheles* mosquitoes, respectively, collected in Queensland, Australia in the 1960s [40,41]. Antibodies against Trubanaman virus have been detected in humans and domestic and wild animals in different parts of Queensland, and the virus is suspected to cause arthritis/rash in humans [42].

Viruses of the species *Batama orthobunyavirus* (Batama virus) and *Tete orthobunyavirus* (Bahig, Matruh, Tete, Tsuruse, Weldona viruses) comprise the Tete serogroup [1,2,32]. Together with Bakau virus (Bakau serogroup) and Estero Real virus (Patois serogroup), Tete serogroup viruses are the only currently known classified orthobunyaviruses transmitted by ticks. We sequenced Bahig, Matruh, and Tete viruses. Bahig and Matruh viruses, first discovered in 1966 and 1961, respectively [43], have been repeatedly isolated from birds and ticks collected in Egypt and Italy; successful isolations have also been made from birds trapped in Cyprus [54,55,56]. Tete virus, the prototype virus of the serogroup, was originally isolated from a spotted-backed weaver bird (*Ploceus cucullatus*) collected in South Africa in 1959 [44].

Finally, we sequenced Umbre virus, a virus of the Turlock serogroup that is represented by viruses belonging to the two species *M’Poko orthobunyavirus* (M’Poko virus, Yaba-1 virus) and *Turlock orthobunyavirus* (Lednice virus, Turlock virus, Umbre virus) [1,2]. Turlock serogroup viruses are distributed in Africa, Asia, Europe, and Northern and South America. Umbre virus was initially isolated from *Culex bitaeniorhynchus* mosquitoes collected in Poona (today Pune), India in 1955 [44]. Additional virus isolates were obtained from *Culex* mosquitoes and birds in India and Malaysia. Anti-Umbre virus antibodies could be detected in sera collected from wild birds and sentinel chickens from Malaysia [35].

In addition, we determined coding-complete genomic sequences of two unclassified members of the family *Bunyaviridae*, Tataguine virus and Witwatersrand virus (Table 1). Tataguine virus was initially recovered from pooled *Culex* and *Anopheles* mosquitoes collected in Senegal in 1962 [57]. This virus is widespread in Africa as evidenced by isolation from areas of today’s Cameroon, Central African Republic, Ethiopia, Nigeria, and Senegal [45,58,59,60,61,62,63,64]. Tataguine virus is a known human pathogen; infected patients present with fever, headache, rash, and joint pain [63,64,65]. Witwatersrand virus was originally isolated from *Culex* mosquitoes collected in Germiston, South Africa, in 1958 [46]. Additional isolates were obtained from *Culex* mosquitoes, sentinel hamsters, and various rodents sampled in Mozambique, South Africa, and Uganda. Antibodies to Witwatersrand virus also have been detected in human sera, although the virus has not been associated with human disease [66].

Our data unequivocally identify both viruses as members of the genus *Orthobunyavirus* and largely confirm the previously deduced relationships of the remaining 15 viruses using serological assays.

## 2. Materials and Methods

### 2.1. Viral Genomic RNA Isolation and Library Preparation

Orthobunyairuses were obtained from the Russian State Collection of Viruses in the form of lyophilized infected suckling mouse brains (Table 1). Total RNA was isolated from vials with 1 mL of TRI Reagent (Molecular Research Center, Cincinnati, OH, USA). Total RNA was additionally purified using the RNeasy MinElute Cleanup Kit (Qiagen, Hilden, Germany) followed by ribosomal RNA depletion using the GeneRead rRNA Depletion Kit (Qiagen) according to the manufacturers’ instructions. Purified RNA was reverse-transcribed with RevertAid Reverse Transcriptase (Thermo Fisher Scientific, Grand Island, NY, USA) using hexameric random primers (Promega, Madison, WI, USA). First strand cDNA was converted to double-stranded cDNA using the NEBNext Second Strand Synthesis Module (New England BioLabs, Ipswich, MA, USA) according to the manufacturer’s instructions. Resulting dsDNA was used to prepare next-generation sequencing libraries using the TruSeq DNA LT Library Prep Kit (Illumina, San Diego, CA, USA). A paired-end 250-bp protocol was used for sequencing indexed libraries on an Illumina MiSeq instrument.

### 2.2. Bioinformatic and Phylogenetic Analyses

Primary analysis of sequencing data and *de novo* genome assembly were performed with CLC Genomics Workbench 7.0 (CLC bio, Waltham, MA, USA). Open reading frame (ORF) analysis and general work with assembled contigs were performed using the Lasergene 11.0.0 (DNAStar, Madison, WI, USA) software package. After *de novo* assembly of trimmed reads, BLASTx (BLAST, basic local alignment search tool, open-source software) analysis was performed against orthobunyaviral sequences, and matching contigs were extracted. All resulting contigs contained parts of non-coding terminal regions and full-length ORFs corresponding to their matches in the BLASTx search. Identified ORFs were translated, and the resulting amino acid (aa) sequences were used in further analyses. Deduced aa sequences of the proteins encoded by sequenced orthobunyaviral genome segments and corresponding sequences of selected representatives of already characterized orthobunyaviruses were aligned using all available multiple sequence alignment methods implemented on M-Coffee server [67] and only columns with a score of 5 or higher were retained. N, glycoprotein precursor polyprotein, and RdRp final alignment lengths were 240, 1412, and 2331 aa residues, respectively. Most suitable models of protein evolution were predicted with open-source ProtTest 3.2 for three alignments [68].

Phylogenetic trees were inferred using MrBayes 3.2.4 [69] under LG + G + I model for N and LG + G + I + F model for glycoprotein precursor polyprotein and RdRp alignments, with 1,000,000 generations and a 25% burn-in value. Maximum likelihood (ML) phylogenies were inferred using MEGA6 with the same protein evolution models and 1000 bootstrap replicates. Consensus trees were visualized with TreeGraph 2.4 [70].

Signal peptide cleavage sites of orthobunyaviral glycoprotein precursors were determined from deduced aa sequences using SignalP 4.1 Server [71]. Transmembrane domains of glycoprotein precursors were predicted using the same sequences and open-source TMHMM Server v 2.0 [72]. Putative *N*-glycosylation sites were determined with the open-source NetNGlyc 1.0 Server [73].

## 3. Results

**Figure 1:**
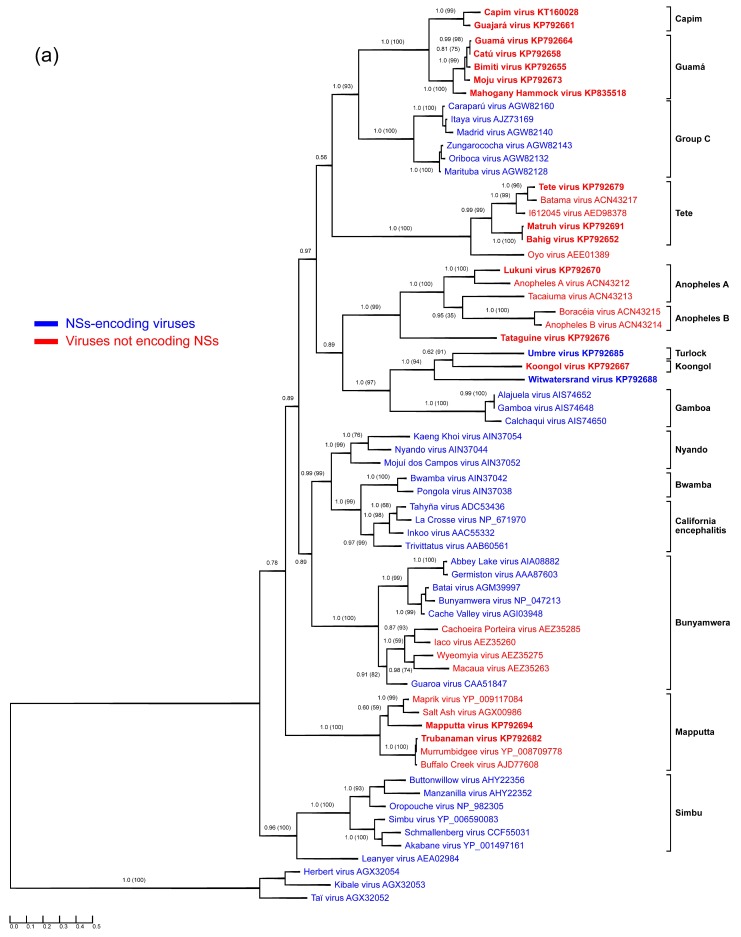

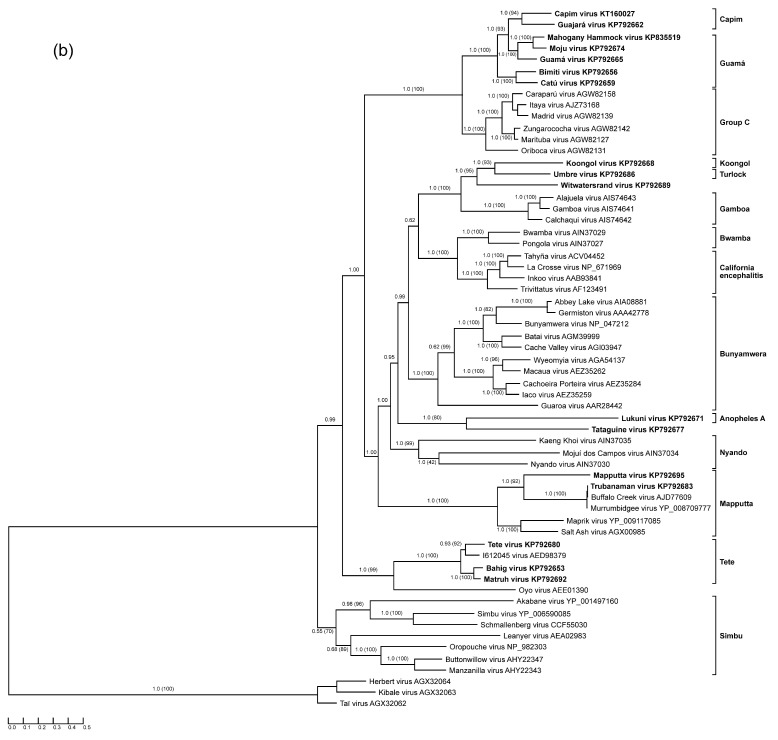

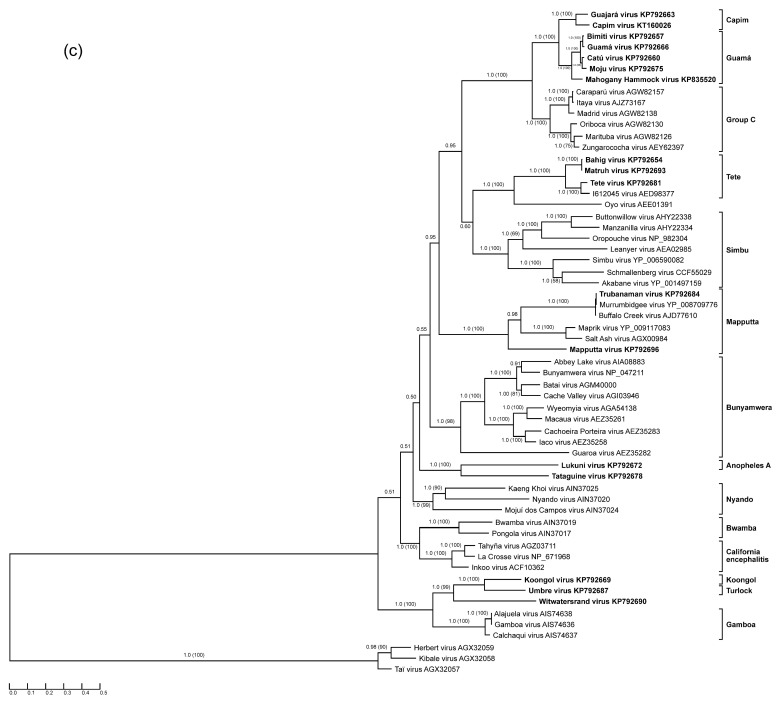
Protein sequence-based phylogenies of orthobunyaviruses. Phylogenies were inferred for (**a**) N; (**b**) glycoprotein precursor polyprotein; and (**c**) L proteins using Bayesian and Maximum Likelihood analyses. The LG + I + G model of amino acid (aa) substitution was used for inferring the N protein phylogeny, whereas the LG + I + G + F model was used to investigate glycoprotein precursor polyprotein and L protein phylogenetic relationships. Numbers represent Bayesian posterior probabilities (Maximum Likelihood bootstrap values). “Herbeviruses” (Herbert virus, Kibale virus, and Taï virus) [5,6] were used to root the phylograms. Trees are drawn to scale measured in substitutions per site. In (a), viruses that encode NSs proteins are marked in blue, whereas viruses that do not are marked in red. Viruses studied in the present work are depicted in bold. GenBank accession numbers and serogroups are to the right of virus names.

Segment-specific ORF-based phylogenies of the genus *Orthobunyavirus*, including previously and newly characterized viruses, are presented in Figure 1.

### 3.1. Anopheles A Serogroup

Genomic data of the Anopheles A serogroup orthobunyaviruses were thus far limited to S segment sequences of Anopheles A and Tacaiuma viruses [74]. Here, we expanded this sequence space by adding the complete coding sequences of Lukuni virus. Based on the obtained genomic data, Lukuni virus is closely related to Anopheles A virus (71.5% aa nucleocapsid sequence identity) and is more distantly related to Tacaiuma virus (53.9% nucleocapsid sequence identity).

### 3.2. Guamá and Capim Serogroups

All Guamá serogroup members studied in the present work (Bimiti, Catú, Guamá, Mahogany Hammock, Moju viruses) clustered together in all three phylogenetic trees (Figure 1), and formed a monophyletic group along with Capim [34,35] and Group C serogroup viruses. The Group C serogroup includes four species: *Caraparu orthobunyavirus* (Apeú, Bruconha, Caraparú, Ossa, Vinces viruses), *Madrid orthobunyavirus* (Madrid virus), *Marituba orthobunyavirus* (Gumbo Limbo, Marituba, Murutucú, Nepuyo, Restan, Zungarococha viruses), and *Oriboca orthobunyavirus* (Itaquí, Oriboca viruses) [1,2]. Group C serogroup viruses were originally thought not to be related to Guamá group viruses, but a low level of HI cross-reactivity between them links these groups of viruses [47].

The measured divergence of Guamá serogroup viruses is somewhat inconsistent among phylogenies inferred for different viral proteins (Figure 1). For instance, based on the phylogenies obtained for N and L proteins, Guamá, Catú, Moju, and Bimiti viruses are more closely related to each other than to Mahogany Hammock virus, although exhibiting different branching orders inside the group. Additionally, analysis of the glycoprotein precursor polyproteins pairs Moju virus with Guamá virus, Mahogany Hammock virus, and Capim serogroup viruses. Taken together, these discrepancies suggest that all examined Guamá group viruses may be reassortants.

The most obvious example of this observation is the relationship between Catú BeH 151 and Guamá BeAn 277 viruses, which were found to be almost identical when compared by their N protein sequences, differing only by two aa (99.2% sequence identity). However, their glycoprotein precursor polyprotein and L protein sequences are more divergent (64.5% polyprotein identity, 95.8% L protein identity). These data are in agreement with original studies that distinguished these two viruses in NT, but not in CF tests [37]. The observed relationships between Catú and Guamá viruses support the idea that one or both of the viruses may be reassortants.

Capim and Guajará viruses fall basal to viruses of the Guamá group phylogenies inferred for N and L proteins, but are placed inside the Guamá serogroup in the glycoprotein precursor polyprotein phylogeny (Figure 1). Capim and Guajará viruses are one-way reactive with Guamá, and Catú and Guamá viruses, respectively, in NT but not in CF assays. Interestingly, no reactivity between Capim and Guajará viruses was found in NT [35]. Bushbush virus, another member of the Capim serogroup, has similar cross-reactivity in NT but not in the CF assay with Bimiti and Catú viruses of the Guamá serogroup [35]. The observed phylogenetic and antigenic relationships between Guamá and Capim serogroup viruses might indicate that their ancestors were inter-group reassortant viruses that shared an M segment. If validated by further experiments and analyses, this finding may be of significance for orthobunyavirus taxonomy, as thus far reassortment has only been observed among viruses of the same orthobunyaviral species and this restriction has been used as one of the official orthobunyavirus species demarcation criteria [2].

### 3.3. Mapputta Serogroup

The obtained genomic sequences of Mapputta virus MRM186 is more than 99% identical to the sequences of the same isolate reported previously [24], and all identified nucleotide (nt) substitutions are synonymous. These substitutions likely arose due to different passaging and maintenance procedures. Our phylogenetic analysis placed Trubanaman virus firmly inside the Mapputta serogroup and revealed its close relationship to two previously characterized viruses: Buffalo Creek virus and Murrumbidgee virus [24,52,53]. The N protein divergence of Buffalo Creek, Murrumbidgee, and Trubanaman viruses does not exceed 2.1%, suggesting that Buffalo Creek and Murrumbidgee viruses are different isolates of Trubanaman virus rather than distinct viruses. An increasing need for further characterization of this group of viruses is indicated by evidence that Buffalo Creek virus [52] and Trubanaman virus are suspected as human pathogens [42].

### 3.4. Tete Serogroup

Sequence information for this serogroup was limited to the S segment of Tete and Batama viruses [74] and a partial Weldona virus M segment sequence. We provide complete coding sequences for three Tete serogroup viruses: Tete, Bahig, and Matruh viruses. Interestingly, the S segment sequence of Tete virus SAAn 3518 obtained here differs slightly from that previously reported [74]. The conflicting region is located closer to the C-terminus of the N protein and, in our case, is represented by a characteristic amino acid motif (G_158_–S_164_) found in all other N protein sequences belonging to Tete group viruses, but not in the Tete virus SAAn 3518 sequence reported earlier. Our analyses reveal Bahig and Matruh viruses to be closely related, with 98.8%, 99.1%, and 87.2% aa identities among their N, L, and glycoprotein precursor polyprotein sequences. This observed relationship is in agreement with the results of CF tests, which showed that these viruses were practically indistinguishable, and HI tests, which proved them to be easily distinguishable [35]. The obtained phylogenetic tree topology and branching order of Tete serogroup viruses (Figure 1) is consistent among the three segments.

Two unclassified bunyaviruses, I612045 virus (GenBank HM627179-81) and Oyo virus (GenBank HM639778-80) form a monophyletic group with Tete serogroup viruses, with Oyo virus falling basal to the other viruses in this group. While the measured nt and aa identities of I612045 virus with other orthobunyaviruses clearly indicate its taxonomic status as a member of Tete serogroup, Oyo virus is indeed distinct and may represent a distinct species in the genus *Orthobunyavirus.*

### 3.5. Koongol and Turlock Serogroups

Here we report coding-complete sequences of all three genomic segments of Koongol and Umbre viruses. The sequence of the Umbre virus IG1424 M segment is more than 99% identical to a previously published M segment of the same isolate [75]. Phylogenetic analysis placed Koongol and Umbre viruses along with unclassified Witwatersrand virus (see below) into a monophyletic group regardless of the protein assayed for tree reconstruction. These viruses also share a last common ancestor with Gamboa serogroup viruses, which are exclusively distributed in North and South America.

Genomic sequence information for the Turlock serogroup was thus far limited to partial Umbre virus M segment sequences. We expanded the sequence space of this serogroup by determining the coding-complete Umbre virus genome sequence and confirmed the relationship of Umbre virus to Kongool and Witwatersrand viruses (Figure 1).

### 3.6. Tataguine and Witwatersrand Viruses (Unassigned Bunyaviruses)

We present sequence information for Tataguine virus, the closest relative of which appears to be Lukuni virus, with 47.5% aa identity for N, 35.5% aa identity for glycoprotein precursor polyprotein, and 53.5% aa identity for L. Anopheles A and Anopheles B group viruses, along with Tataguine virus, form a monophyletic group with Tataguine virus at its base. Our data indicate that Tataguine virus may have to be assigned to a new species in the genus *Orthobunyavirus.*

As mentioned above, based on the obtained phylogenies, Witwatersrand virus clusters together with Umbre (Turlock serogroup) and Koongol (Koongol serogroup) viruses in the same branching order independent of the analyzed protein. The closest relative of Witwatersrand virus is Koongol virus (48.5% to 59.6% aa identities for the three proteins), indicating that Witwatersrand virus should be classified as an orthobunyavirus.

### 3.7. Characteristics of S Segments: N and NSs proteins

The N proteins of bunyaviruses encapsidate viral RNA and are major CF determinants [3]. The lengths of the N proteins of the examined orthobunyaviruses range from 234 aa residues for Koongol virus (Kongool serogroup) to 258 aa for Bahig, Matruh, and Tete viruses (Tete serogroup) (Table 2). Our studies confirm that Tete serogroup viruses possess the longest N proteins in the genus *Orthobunyavirus* with unique extensions predominantly located at the amino termini [74].

**Table 2:** Protein lengths of examined orthobunyaviruses (in amino acid residues)*.

Orthobunyavirus Serogroup Virus Name (Virus Abbreviation)	N	NSs	Glycoprotein Precursor (# of Cysteines)	Gn	NSm	Gc	L
**Anopheles A serogroup**							
Lukuni virus (LUKV)	242	-	1408 (71)	286	168	940	2241
**Capim serogroup**							
Capim virus (CAPV)	235	-	1430 (74)	284	188	947	2252
Guajará virus (GJAV)	235	-	1435 (73)	286	188	948	2252
**Guamá serogroup**							
Bimiti virus (BIMV)	237	-	1443 (73)	284	191	954	2250
Catú virus (CATUV)	237	-	1440 (72)	284	191	952	2250
Guamá virus (GMAV)	237	-	1439 (78)	284	187	952	2250
Mahogany Hammock virus (MHV)	237	-	1436 (75)	284	189	946	2250
Moju virus (MOJUV)	237	-	1435 (75)	284	189	946	2250
**Koongol serogroup**							
Koongol virus (KOOV)	234	-	1105 (57)	284	38	777	2270
**Mapputta serogroup**							
Mapputta virus (MAPV)	236	-	1370 (77)	288	161	910	2241
Trubanaman virus (TRUV)	237	-	1371 (71)	286	164	908	2242
**Tete serogroup**							
Bahig virus (BAHV)	258	-	1433 (69)	286	179	955	2280
Matruh virus (MTRV)	258	-	1433 (67)	286	179	955	2280
Tete virus (TETEV)	258	-	1432 (68)	286	178	955	2281
**Turlock serogroup**							
Umbre virus (UMBV)	237	79	1466 (72)	284	176	991	2293
**Unassigned**							
Tataguine virus (TATV)	239	-	1446 (73)	287	171	976	2246
Witwatersrand virus (WITV)	245	111	1448 (70)	285	173	974	2288

* Gn, NSm, and Gc lengths were calculated by implying Gn-NSm cleavage at conserved residue R_302_ (Bunyamwera virus). NSm-Gc cleavage was predicted by SignalP 4.1 server.

**Figure 2:**
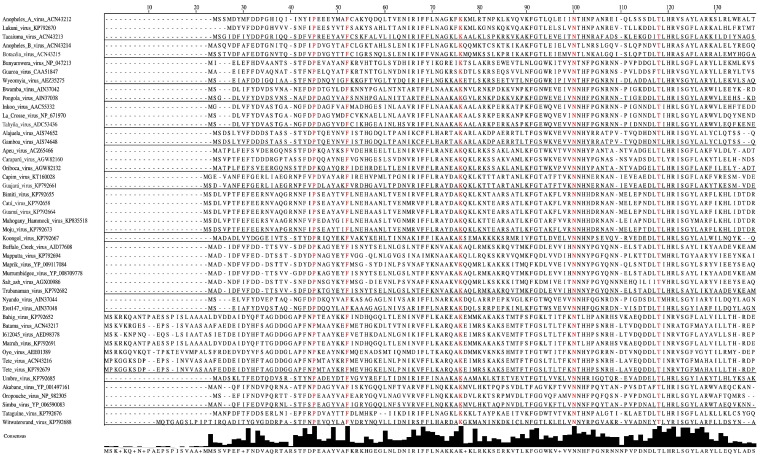

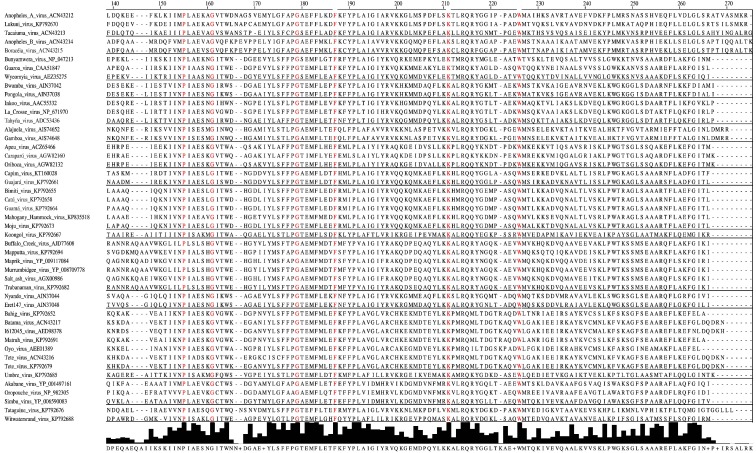
Amino-acid alignment of orthobunyaviral nucleocapsid protein sequences. Alignment was performed on 118 nucleocapsid sequences belonging to different orthobunyaviruses using the Clustal algorithm with default settings as implemented in Jalview 2.9 [76], followed by hiding all but selected representatives of the serogroups. Sites that are strictly conserved among all aligned sequences are depicted in red. Consensus histograms, calculated for all aligned sequences, represent proportions of sites matching corresponding positions of the consensus sequence.

Forty-six aa of the N protein are conserved among the previously determined 51 sequences of orthobunyaviruses from the Bunyamwera, California, Group C, and Simbu serogroups [77]. Our analyses reveal that only 11 aa of these 46 aa are strictly conserved among the N proteins of Anopheles A, Anopheles B, Bunyamwera, Bwamba, California, Capim, Gamboa, Guamá, Group C, Koongol, Turlock, Nyando, Simbu, and Tete serogroup viruses (Figure 2). Five of these 11 aa (F26, P125, G131, K179, W193) are crucial for Bunyamwera virus mini-replicon rescue. Two of those aa, K179 and W193, are likely involved in RNA synthesis, and two other aa residues, P125 and G131, are thought to play a role in ribonucleoprotein packaging [77].

A number of researchers have evaluated whether NSs is involved in the immune response to orthobunyavirus infections. Despite not expressing NSs, Tacaiuma virus (Anopheles A serogroup) antagonizes host interferon (IFN) production through a yet unrecognized mechanism [74] and is associated with human febrile illness [35]. Similarly, Mapputta serogroup viruses, such as Maprik virus and Buffalo Creek virus, do not encode NSs, but are linked to human diseases [24]. Two out of 17 orthobunyaviruses studied in the present work, Umbre virus (Turlock serogroup) and Witwatersrand virus (ungrouped orthobunyavirus), encode NSs proteins of 79 and 111 aa, respectively (previously characterized orthobunyaviruses: 62 (Group C serogroup) to 130 aa (Gamboa serogroup)). Neither of the two viruses has been associated with human disease. In contrast, known human pathogens, such as the febrile disease-causing ungrouped Tataguine virus and Guamá and Catú viruses (Guamá serogroup), do not encode NSs. These findings suggest that the presence or absence of an NSs-encoding ORF alone does not predic human pathogenicity. Additionally, the presence of an NSs-encoding ORF is far less common for orthobunyaviruses than previously thought as only viruses from 8 out of 15 sequenced serogroups do encode this nonstructural protein.

### 3.8. Characterization of M segments: Glycoprotein Precursor Polyprotein and Gn and Gc Proteins Cleavage Products

Each orthobunyavirus M segment contains a single continuous ORF encoding a glycoprotein precursor polyprotein that is cotranslationally cleaved into glycoprotein Gn, the nonstructural protein NSm, and the glycoprotein Gc [78]. Among the polyprotein sequences derived from the sequenced M segments of our study, the Koongol virus glycoprotein precursor polyprotein (1105 aa) is notably smaller than all other glycoprotein precursors of orthobunyaviruses, which range in size from 1370 aa in the case of Mapputta virus to 1448 aa in the case of Witwatersrand virus, Table 1. A strictly conserved arginine residue of the glycoprotein precursor polyprotein sequences of all sequenced orthobunyaviruses (position 302 in the prototype Bunyamwera virus) is believed to mark the cleavage site between Gn and NSm proteins [78]. Therefore, Koongol virus Gn is comparable in size with Gn of other orthobunyaviruses whilst its Gc and NSm proteins are notably shorter.

Regions highly similar to the fusion peptide identified in La Crosse virus glycoprotein precursor polyprotein (positions 1066–1087) are present in the predicted polyproteins of the sequenced orthobunyaviruses of all serogroups. Ten out of twenty-two La Crosse virus fusion peptide aa are strictly conserved across the genus. This finding indicates that all orthobunyavirus Gc glycoproteins have analogous functions and thereby act as class II fusion proteins [79]. Supporting this hypothesis is the finding that the overall topology of orthobunyaviral glycoprotein precursor polyprotein appears to be conserved based on the number of conserved cysteine residues (ranging from 67 in the case of Matruh virus (Tete serogroup) to 78 in the case of Guamá virus (Guamá serogroup)). Once again, Koongol virus (Koongol serogroup) is the outlier with only 57 cysteine residues [80,81,82].

*N*-glycosylation of viral membrane proteins plays a crucial role in correct protein folding and functioning, including receptor binding, membrane fusion, and cell-penetration processes [83]. All three predicted *N*-glycosylation sites of the membrane glycoproteins of Bunyamwera virus (Bunyamwera serogroup) are indeed glycosylated. Glycosylation of Bunyamwera virus Gn’s N60 site is essential for correct protein folding of both Gn and Gc [84]. Interestingly, this glycosylation site is highly conserved among almost all previously sequenced orthobunyaviruses, with the notable exception of Maprik virus (Mapputta serogroup) [24]. NetNGlyc 1.0 server predicted this glycosylation site to be present in the Gn proteins of all viruses sequenced in this study, with the exception of Lukuni virus (Anopheles A serogroup) and ungrouped Tataguine viruses. In general, glycosylation site locations were moderately conserved in orthobunyaviruses belonging to the same serogroup, but were not consistent among all viruses of the genus *Orthobunyavirus*. Finally, with the exception of Koongol virus, transmembrane prediction using hidden Markov models (TMHHM) 2.0 generally predicted the same distribution and type of transmembrane regions for the glycoprotein precursor polyprotein sequences of the analyzed viruses, which included two transmembrane domains in Gn, three in NSm, and one in Gc. The Koongol glycoprotein precursor polyprotein has four predicted transmembrane regions, lacking two domains usually located at the C-terminal half of NSm.

### 3.9. Characterization of L Segments: RNA-Dependent RNA Polymerase

The L sequence aa lengths of the studied orthobunyaviruses are comparable to those of previously studied viruses, ranging from 2241 aa in the case of Lukuni virus to 2293 aa in the case of Umbre virus (Table 2). Tete group (Bahig, Matruh, and Tete viruses) L (2280–2281 aa) possesses a serogroup-characteristic 24 aa insertion (E2185–E2208) at the C terminus. Umbre and Witwatersrand virus L possess several aa at the very end of the C terminus that are not conserved among other viruses. All analyzed L sequences have the same well-conserved topology, consisting of four distinct regions with readily distinguishable RNA-dependent RNA polymerase motifs pre-A to E inside the POL III block. The proposed site-active domain SDD1163–5 of Bunyamwera virus [85] is strictly conserved among all previously and newly characterized orthobunyaviruses.

## 4. Discussion

The family *Bunyaviridae* was originally established to group viruses that produce morphologically similar and often serologically cross-reactive virions [86]. Largely non-sequence-based efforts [9,10,11,12,13,14] further grouped the members of this family into various serogroups [87,88], which were later assembled in higher-order taxa, *i.e.*, the currently accepted genera *Hantavirus*, *Nairovirus*, *Orthobunyavirus*, *Phlebovirus*, and *Tospovirus* [2,89]. Recent large-scale efforts to sequence the genomes of bunyaviruses and other segmented negative- or ambisense-stranded RNA viruses revealed that numerous novel bunyaviruses cannot be assigned to the five existing genera and that a plethora of viruses that are clearly related to bunyaviruses do not fit their classical definition of being trisegmented, *i.e.*, arenaviruses, emaraviruses, tenuiviruses, and Mourilyan virus [1,5,23,28,90,91,92,93,94,95,96,97,98,99,100,101,102,103,104,105,106,107,108,109]. The International Committee on Taxonomy of Viruses (ICTV) *Bunyaviridae* Study Group has therefore recently initiated discussions on a taxonomic re-evaluation of the entire bunyavirus-like supergroup with the ultimate goal to establish a novel taxonomy that adequately reflects the phylogenetic relationships of all these viruses. Unfortunately, the genomic “sequence space” of the supergroup is still very limited, thereby impeding those efforts.

Within the classic orthobunyavirus group, genomic sequence information was limited to viruses of 10 of 18 serogroups [15,16,17,18,19,20,21,22,23,24,25,26,27,28]. Our work expands this sequence space by adding coding-complete genomic information on viruses of an additional five serogroups. Our efforts largely confirm the relationships of the studied viruses that had been established previously by non-sequence-based (serological) techniques. Despite the high genetic diversity of the orthobunyaviruses, reflecting their wide geographic distribution and variety of ecological features, the viruses of each serogroup are grouped together within the appropriate lineage on the three phylogenetic trees. Since the members of the *Bunyaviridae* family possess segmented genomes, the phenomenon of segment reassortment plays a significant role in their evolution [110]. Earlier, it was shown that many members of the genus *Orthobunyavirus* are intra-group reassortants. Our data show that all examined viruses of the Guamá serogroup are in all likelihood genome segment reassortants. Furthermore, viruses of the Guamá and Capim serogroups form a monophyletic lineage on the tree inferred for the glycoprotein polyprotein precursor protein (M segment), suggesting inter-group reassortment of M segments in their natural history. Another important observation that could be made is the classification of Witwatersrand virus and Tataguine virus as likely members of two new novel species in the genus *Orthobunyavirus*.

Finally, our data show that presence of an ORF encoding an NSs protein is not a universal feature for orthobunyaviruses. Among the viruses of 15 orthobunyavirus serogroups for which genomic data are now available, only viruses of eight serogroups along with Witwatersrand virus encode NSs proteins. Therefore, the presence or absence of NSs protein should not be considered as a taxonomic characteristic of the genus *Orthobunyavirus*, but may be important for differentiating pathogenic from nonpathogenic viruses.

Our analyses advance the overall resolution of orthobunyavirus phylogeny. Although sequence information for the 3′ and 5′ genomic segment termini could not obtained in this study, we are confident that the determined phylogenetic placement on the phylogenetic tree of all viruses studied here will hold. Complementation of our results by genomic sequences of viruses from the remaining unsequenced orthobunyavirus serogroups should allow official taxonomic re-organization of the genus *Orthobunyavirus*.
